# Non-invasive assessment of inter-and intrapatient variability of integrin expression in metastasized prostate cancer by PET

**DOI:** 10.18632/oncotarget.8611

**Published:** 2016-04-06

**Authors:** Ambros J. Beer, Sarah M. Schwarzenböck, Niko Zantl, Michael Souvatzoglou, Tobias Maurer, Petra Watzlowik, Horst Kessler, Hans-Jürgen Wester, Markus Schwaiger, Bernd Joachim Krause

**Affiliations:** ^1^ Department of Nuclear Medicine, Klinikum rechts der Isar, Technische Universität München, 81675 Munich, Germany; ^2^ Department of Urology, Klinikum rechts der Isar, Technische Universität München, 81675 Munich, Germany; ^3^ Institute for Advanced Study (IAS) and Center of Integrated Protein Science (CIPSM), Department Chemie, Technische Universität München, 85747 Garching, Germany; ^4^ Institute for Radiopharmaceutical Chemistry, Technische Universität München, 85748 Garching, Germany; ^5^ Current address: Department of Nuclear Medicine, Ulm University, 89081 Ulm, Germany; ^6^ Current address: Department of Nuclear Medicine, Rostock University Medical Centre, 18057 Rostock, Germany; ^7^ Current address: Department of Urology, Klinikum Konstanz, 78464 Konstanz, Germany

**Keywords:** prostate cancer, integrins, αvβ3, angiogenesis, PET

## Abstract

**Purpose:**

Due to the high expression of the integrin αvβ3 not only on endothelial cells, but also on mature osteoclasts and prostate cancer cells, imaging of osseous metastases with αvβ3-targeted tracers seems promising. However, little is known about the patterns of αvβ3-expression in metastasized prostate cancer lesions in-vivo. Thus we evaluated the uptake of the αvβ3-specific PET tracer [^18^F]Galacto-RGD for assessment of bone metastases in prostate cancer patients.

**Results:**

[^18^F]Galacto-RGD PET identified 58/74 bone-lesions (detection rate of 78.4%) and lymph node metastases in 2/5 patients. The SUV_mean_ was 2.12+/−0.94 (range 0.70–4.38; tumor/blood 1.36+/−0.53; tumor/muscle 2.82+/−1.31) in bone-lesions and 2.21+/−1.18 (range 0.75–3.56) in lymph node metastases. Good visualization and detection of bone metastases was feasible due to a low background activity of the surrounding normal bone tissue.

**Methods:**

12 patients with known metastasized prostate cancer according to conventional staging (including bone-scintigraphy and contrast-enhanced CT; median PSA 68.63 ng/ml, range 3.72-1935) were examined with PET after i.v.-injection of [^18^F]Galacto-RGD. Two blinded nuclear-medicine physicians evaluated the PET-scans in consensus concerning lesion detectability. Volumes-of-interest were drawn in the PET-scans over all metastases defined by conventional staging (maximum of 11 lesions/patient), over the left ventricle, liver and muscle and standardized-uptake-values (SUVs) were calculated.

**Conclusions:**

Our data show generally elevated uptake of [^18^F]Galacto-RGD in bone metastases from prostate cancer with a marked inter- and intrapatient variability. While [^18^F]Galacto-RGD PET is inferior to bone scintigraphy for detection of osseous metastases, it might be valuable in patient screening and monitoring of αvβ3-targeted therapies due to the high variability of αvβ3-expression.

## INTRODUCTION

The integrin αvβ3 is an interesting target for specific therapies in oncology and as a new prognostic factor, as it is highly expressed on activated endothelial cells during angiogenesis and plays an important role in the regulation of tumor growth, local invasiveness and metastatic potential [1, 2]. Moreover, αvβ3 is expressed in prostate cancer cells but not in normal prostate cells [3]. Furthermore, the risk of bone metastasis in advanced prostate cancer is correlated to integrin-mediated interaction of metastatic cancer cells and bone microenvironment [4]. Preclinical studies show that αvβ3 integrin mediates the adhesion of prostate cancer cells to ECM components of the bone such as osteopontin [5, 6]. This is in accordance with the finding that prostate cancer cell lines derived from bone metastases uniformly express αvβ3 [7]. Αv-integrins also promote survival of prostate cancer cells in bone [4] and increase the aggressiveness of prostate cancer cells [8]. Moreover, the integrin αvβ3 mediates osteopontin triggered proliferation of castration resistant prostate cancer cells in bone [9]. Interaction of αvβ3 as well as αvβ5 with osteopontin is involved in bone turnover by osteoblasts and osteoclasts [10, 11]. There is also growing evidence that integrin crosstalk with growth factor cytokines could have important implications for tumor metastasis and drug resistance [8]. In summary, integrins αvβ3 and αvβ5 promote metastasis of prostate cancer cells to bone in each step of the metastatic process [12–15]. Due to these findings, there is increasing interest in using antagonists of αvβ3 for specific tumor therapy, like the cyclic peptide Cilengitide^®^ [16–20]. There is evidence for the effectiveness of this approach as blockade of αvβ3 reduces osteoclast recruitment and bone lysis initiated by metastatic cancer cells [21]. Moreover, a clinical trial in metastasized prostate cancer patients showed moderate efficacy of Cilengitide^®^ in this setting with limited toxicity [17]. Thus, imaging of integrin expression might provide relevant information on patient prognosis and risk for metastatic spread especially to the bone in prostate cancer patients and might be valuable within the context of integrin-specific therapies.

However, despite the vast preclinical data on the role of integrins in metastasized prostate cancer, little is yet known about the pattern of αvβ3 expression *in-vivo* in patients with metastasized prostate cancer. One of the reasons might be, that examinations of the intact integrin αvβ3 are difficult in humans, as most antibodies work best on fresh frozen tissue, obviating retrospective analyses in paraffin embedded specimens. Moreover, in general immunohistochemistry can only show parts of the tumor, which might bias the interpretation, because tumors might be heterogeneous and samples for immunohistochemistry might not necessarily have been taken from representative areas. Molecular imaging on the other hand has the potential to show specific biological properties of tissues *in-vivo* as a whole and also in several different tumor sites within the body in one session, even in cases where collecting samples for immunohistochemistry is difficult, like in our patient collective [22]. Imaging of αvβ3 expression might therefore help to elucidate the complex role of this integrin in prostate cancer patients. We have developed the αvβ3 specific tracer [^18^F]Galacto-RGD for positron emission tomography (PET) imaging [23]. It has already been demonstrated, that [^18^F]Galacto-RGD PET allows for specific imaging of αvβ3 expression in tumor xenografts as well as in patients [24–26]. A significant correlation of αvβ3 expression and [^18^F]Galacto-RGD uptake has been proven preclinically and clinically [27–30]. We now report for the first time on the specific use of [^18^F]Galacto-RGD PET in advanced prostate cancer patients. The goal of our study was to analyse the uptake patterns of [^18^F]Galacto-RGD in metastatic prostate cancer lesions, in order to evaluate the potential of imaging of αvβ3 expression with PET for future applications in this patient cohort, like non-invasive assessment of integrin for prognostic stratification or screening of patients before αvβ3 targeted therapies.

## RESULTS

### Quantitative data on [^18^F]Galacto-RGD uptake in malignant lesions

**Figure 1:**
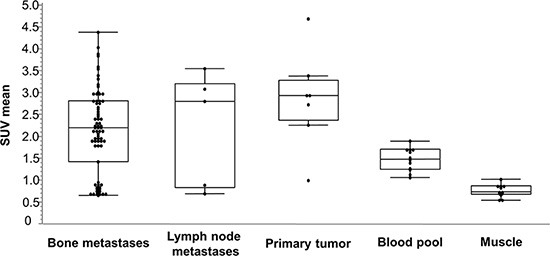
Box-Whisker-Plots of the results of SUVmean measurements for lesions (bone metastases, lymph node metastases and primary tumor), muscle and blood pool

The results of the SUV measurements for tumors, muscle and blood pool are summarized in Figure 1. The SUV_mean_ in bone metastases (*n* = 74) was 2.1 ± 0.9 (range 0.7–4.4), in lymph node metastases (*n* = 5) 2.2 ± 1.2 (range 0.8–3.6) and in primary lesions in the prostate (*n* = 7) 2.9 ± 1.0 (range 1.0–4.7).

In background tissue, SUV_mean_ in blood was 1.5 ± 0.3 and in muscle 0.8 ± 0.1.

This resulted in mean tumor-to-blood/tumor-to-muscle ratios for bone metastases of 1.4 ± 0.5/2.8 ± 1.3, for lymph node metastases of 1.5 ± 0.9/3.2 ± 2.1 and for primary tumors of 2.0 ± 0.8/3.9 ± 1.8.

### Qualitative analysis of [^18^F]Galacto-RGD uptake and detection rate for metastatic lesions

**Figure 2:**
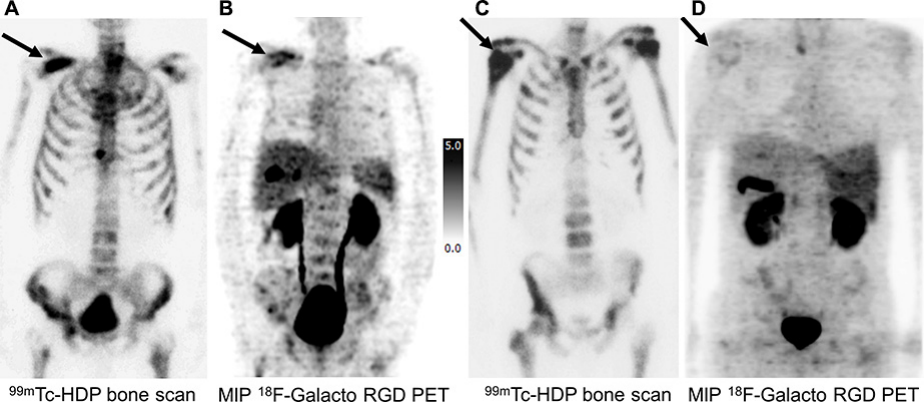
Imaging examples of a comparison of bone metastases in [^99m^Tc]HDP Bone Scan (A, C) and MIP [^18^F]Galacto-RGD PET (B, D) in two prostate cancer patients (**A, B**) 70 year old patient with bone metastases from prostate cancer under antiandrogen therapy and a PSA value of 55.26 ng/ml (initial staging cT3, cN0, cM1; Gleason score 8): [^99m^Tc]HDP Bone Scan (A) revealed an intense uptake in disseminated bone metastases (arrow indicating an osseous metastasis of the right scapula), which also showed a moderately to intensely elevated uptake in [^18^F]Galacto-RGD PET (B) in the majority of lesions. (**C, D**) 61 year old patient with bone metastases from prostate cancer under antiandrogen therapy and a PSA value of 175 ng/ml (initial staging: pT3a, pN0, cM0, G2; Gleason score 8): [^99m^Tc]HDP Bone Scan (C) revealed an intense uptake in disseminated bone metastases (arrow indicating a osseous metastasis of the right humerus). In [^18^F]Galacto-RGD PET (D), however, only faint or no uptake was seen in the bone metastases. These examples demonstrate, that despite very similar patterns of metastatic spread in bone scintigraphy, the findings in [^18^F]Galacto-RGD PET can be totally different. Note physiological uptake of [^18^F]Galacto-RGD in the liver, the spleen as well as in the gallbladder and in the urinary system.

In total, clinical staging including bone scintigraphy and CT when available showed 74 bone metastases in 12 patients. In the static emissions scans of the [^18^F]Galacto-RGD PET scan, detection of bone metastases was feasible in all patients, indicating a patient based detection rate of 100%. In a lesion based analysis 58 of the 74 bone lesions could be identified as areas with increased tracer uptake. In general the uptake was slightly to moderately elevated, however due to the physiologically very low background activity of [^18^F]Galacto-RGD in the bone, also areas with low uptake were identifiable in the skeleton (Figure 2). Physiological uptake of [^18^F]Galacto-RGD is seen in the liver, the spleen and intestine. The excretion via the urinary system is similar to the excretion of [^18^F]FDG. Unlike [^18^F]FDG, a minor amount of [^18^F]Galacto-RGD is also excreted by the hepatobiliar pathway, so in some patients tracer uptake is seen in the gallbladder. Another difference to [^18^F]FDG is that [^18^F]Galacto-RGD does not cross the blood-brain-barrier and does not show general muscle uptake or cardiac uptake.

**Figure 3:**
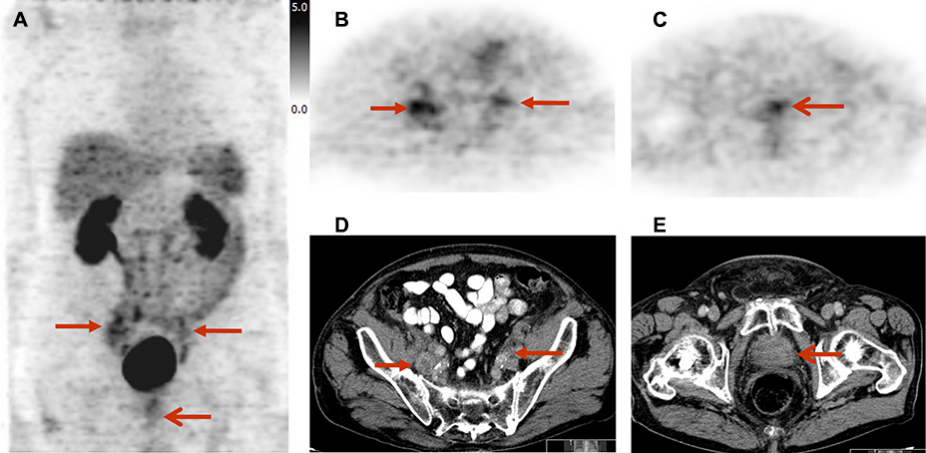
Imaging example of lymph node metastases and primary tumor site in a 82 year old patient with prostate cancer under androgen deprivation therapy (initial staging cT3a, cN1, cM1; Gleason score 7; PSA 146.2 ng/ml): lymph node metastases (B, D) show a slightly to moderately elevated, heterogeneous uptake of [^18^F]Galacto-RGD; the primary tumour site (C, E) shows a slightly elevated uptake Due to a generally higher level of background activity in the intestine and urinary system, detection of tracer uptake in lymph node metastases was more difficult. MIP [^18^F]Galacto-RGD PET (**A**), PET (**B**) and CT (**D**) showing lymph node metastases, PET (**C**) and CT (**E**) showing primary local tumor site. Arrows indicating lymph node metastases and primary local tumor site.

Five patients presented with lymph node metastases. Of these, 2 patients could also be identified by [^18^F]Galacto-RGD PET due to increased tracer uptake, but also within these patients uptake was heterogeneous with positive and negative lymph node metastases within one patient (Figure 3). Due to the use of PET only and the generally higher level of background activity in the abdomen, detection of tracer uptake in lymph nodes was more difficult compared to the bone.

Seven patients presented with either the primary tumour still in-situ (*n* = 4) or local recurrence (*n* = 3). Of these, in 6 patients an elevated uptake could be identified in the prostate region, although uptake again was generally moderate and detection was impaired by physiological tracer excretion via the bladder.

### Correlation [^18^F]Galacto-RGD uptake with clinical data and PSA value

Concerning patterns of [^18^F]Galacto-RGD uptake in bone metastases, 8 patients with 50 lesions were under androgen deprivation therapy (group 1), while 4 patients with 24 lesion were at the time of PET without antihormonal therapy (group 2). There was no significant difference in the uptake patterns between the two groups: group 1 mean SUV_mean_ ± std-dev.: 2.12 ± 1.07; group 2 mean SUV_mean_ ± std-dev.: 2.13 ± 0.46 (*p* > 0.05).

Correlation of PSA values and [^18^F]Galacto-RGD uptake revealed no clear trend in this patient population. Taking all values including one outlier with a very high PSA value of 1935 ng/ml, a significant but only very weak inverse correlation of PSA and SUV_mean_ was notable (Spearman's *r* = −0.261; *p* = 0.0258). Without that outlier, the inverse correlation was highly significant but only with a moderate correlation coefficient (Spearman's *r* = −0.503; *p* = 0.0001). However, these results are difficult to interpret due to the small patient sample and the inhomogeneity of the patient group.

## DISCUSSION

In this study we could successfully analyze the patterns of [^18^F]Galacto-RGD uptake in prostate cancer metastases. Although the proof-of-concept design of this investigation did not allow for immunohistochemical crossvalidation of tracer uptake, we successfully demonstrated the specificity of [^18^F]Galacto-RGD binding and correlation of tracer uptake with αvβ3-integrin expression in various previous clinical studies. In this study on prostate cancer patients, we observed generally elevated but highly heterogeneous αvβ3 expression with marked intra- and interpatient variability. Due to this variability, imaging of αvβ3 expression in prostate cancer with PET might be valuable for patient screening and monitoring in the context of αvβ3-targeted therapies and potentially for prognostic assessment.

Our results demonstrated [^18^F]Galacto-RGD uptake in bone metastases from prostate cancer as expected, as the role of the integrin αvβ3 in metastatic spread to the bone is well established. Nonetheless, examinations of integrin αvβ3 expression *in-vivo* and especially in patients are scarce. Thus the information provided by our study contributes to the growing literature about integrin expression in human prostate cancer. Tracer uptake in metastases was very heterogeneous with a high intra- and inter-patient variety within the patient sample, suggesting varying levels of integrin expression. A correlation between [^18^F]Galacto-RGD uptake and αvβ3 expression has already been shown preclinically as well as clinically, αvβ3 has been recognized to be an important player in tumor biology and angiogenesis [27]; however, specificity against other subtypes such as α5β1 is not fully understood until now [31]. Nevertheless, these results corroborate data from clinical, preclinical and in-vitro studies on the importance of αvβ3 for tumor spread and especially metastatic spread to the bone [32–34]. This also points out that the role of αvβ3 in tumor metastasis is very complex and that αvβ3 expression either is not mandatory for metastasis formation in every lesion, or that αvβ3 expression can be lost after initial metastatic spread during the course of metastatic growth. For malignant melanoma, this has already been successfully demonstrated preclinically. It is known that the expression of αvβ3 plays an important role during the transition of melanoma cells from the superficial growth phase to the vertical growth phase [35, 36]. The progression to metastases, however, is more complex and may not be dependent on αvβ3 expression or αvβ3 might be expressed in varying quantities during various stages of metastatic dissemination [37]. The expression of αvβ3 on activated endothelial cell in angiogenesis might be of lesser importance in prostate cancer compared to other cancer types; however, this was not the focus of our study.

Although uptake in bone metastases was only slightly to moderately elevated, also bone metastases with a low uptake could be identified due to a very low background activity of [^18^F]Galacto-RGD within the bone. However, the detection rate still was inferior compared to conventional bone scintigraphy. Again this most likely is due to the fact that the expression of integrin αvβ3 in bone metastases is heterogeneous and a complex phenomenon depending on the respective biological context as discussed before. Concerning the primary tumor, in some patients elevated uptake could be identified in the primary tumor site, however the detection of the primary tumor or local recurrence was not the focus of this study thus no systematic conclusion can be drawn. Concerning lymph node metastases, the detection rate in our small patient sample was low. However, again the evaluation of the use of [^18^F]Galacto-RGD PET for staging was not the focus of our study, but it might be assumed that with respect to staging [^18^F]Galacto-RGD PET seems to be limited due to heterogeneous uptake pattern of lymph node metastases and (compared to bone) higher background activity in the abdomen which hampers detection of lymph node metastases. Further conclusions on the use of [^18^F]Galacto-RGD PET for staging patients with advanced prostate cancer cannot be drawn on the basis of our results due to our small and heterogeneous patient sample and due to the limiting fact that PET only was used. However, detection of lymph node metastases might be improved by the use of combined PET/CT, which facilitates differentiation of tracer uptake in lymph nodes from unspecific uptake in the intestine and ureter.

No differences could be shown with respect to uptake patterns of [^18^F]Galacto-RGD in patients with or without antihormonal therapy; again this might be due to the small and heterogeneous patient sample; on the other hand the influence of antihormonal therapy on tracer uptake is also discussed controversially with respect to other tracers such as [^11^C]/[^18^F]Choline. The correlation analysis of PSA values and [^18^F]Galacto-RGD uptake showed a significant but only weak correlation without revealing a clear trend within this small patient sample.

Potential applications of [^18^F]Galacto-RGD PET in patients with prostate cancer could be the further elucidation of the role of αvβ3 in the context of primary and metastatic disease and as a prognostic marker within this patient population. In this respect, PET imaging with specific tracers provides additional information to immunohistochemistry alone, as multiple lesions in the body can be assessed simultaneously and tumour variability can be analyzed as well [38]. Moreover, [^18^F]Galacto-RGD PET might also be used for follow-up examinations, planning and monitoring of targeted therapies including therapy response assessment, like with αvβ3-specific agents as Cilengitide^®^ [16, 17]. However, with respect to therapy response assessment it has to be considered that uptake of tumor lesions is relatively low potentially influencing the evaluation of therapeutic effects in the setting of targeted therapy in case of only partial response and low drop of quantitative uptake values.

There are limitations to our study. Quantification of αvβ3 expression in tumor specimens was not performed, however, exactly the fact that collecting representative biopsy samples in this patient group is difficult or not possible at all was one major motivation for studying the integrin expression non-invasively with molecular imaging. Furthermore imaging was performed as [^18^F]Galacto-RGD PET only. Hybrid imaging modalities such as combined PET/CT or PET/MRI might improve imaging properties by providing and combining functional and exact morphological information. [^18^F]Galacto-RGD showed an adequate imaging contrast with, however, inhomogeneous tracer uptake. Perspectival, other radiolabelled RGD peptides with higher affinity and therefore enhanced integrin-specific uptake, such as [^68^Ga]TRAP(RGD)_3_ as recently published by Notni et al. [39], might further improve imaging properties by allowing for imaging of low-level integrin expression.

## CONCLUSIONS

While [^18^F]Galacto-RGD PET is inferior to conventional imaging procedures like bone scintigraphy for detection of osseous metastases, it could successfully identify generally elevated αvβ3 expression in bone metastases from prostate cancer, but with a marked inter- and intrapatient variability. Due to this variability, imaging of αvβ3 expression might be valuable in patient screening and monitoring in the context of αvβ3-targeted therapies or as a prognostic factor, which based on these promising first data now has to be proven in further prospective studies.

## MATERIALS AND METHODS

### Radiopharmaceutical preparation

Synthesis of [^18^F]Galacto-RGD was carried out under identical conditions and with identical radiochemical yield and radiochemical purity as described previously [40].

### Patients

The local ethics committee approved the study and informed written consent was obtained from all patients. 12 patients were retrospectively examined with known metastasized prostate cancer according to clinical staging (including [^99m^Tc]HDP bone scintigraphy in all cases and contrast-enhanced CT in 8 cases). The median PSA was 68.63 ng/ml with a range of 3.72–1935 ng/ml. A further inclusion criteria was age over 18 years. Exclusion criteria consisted of impaired renal function (serum creatinine level > 1.2 mg/dl). The patient characteristics are summarized in Table 1.

**Table 1:** Summary of the patient characteristics

Age	TNM at initial staging	Gleason score	PSA (ng/ml)	Antiandrogen Tx	LN metastases	Distant metastases	Prostate bed: local situation
64	pT3a, cN1, cM1	7	4.1	no	no	yes	local recurrence
49	pT3, pN1, cM1	9	3.7	no	yes	yes	local recurrence
78	cT3, cN1, cM1	9	82.0	no	yes	yes	no recurrence
72	cT3, cN0, cM1	7	163.9	no	no	yes	no recurrence
76	pT3b, pN2, cMx	9	14.0	yes	no	yes	local recurrence
63	cT4, cN0, cM1	9	300.0	yes	no	yes	primary tumour
74	cT3, cN1, cM1	9	24.0	yes	yes	yes	primary tumour
61	pT3a, pN0, cMx	8	175.0	yes	no	yes	no recurrence
54	cT3, cN1, cM1	8	1935.0	yes	yes	yes	no recurrence
60	cT3, cN0, cMx	9	23.7	yes	no	yes	primary tumour
70	cT3, cN0, cM1	8	55.3	yes	no	yes	no recurrence
82	cT3a, cN1, cM1	7	146.2	yes	yes	yes	primary tumour

### PET imaging procedure

Imaging was performed with an ECAT EXACT PET scanner (CTI/Siemens, Knoxville). Before injection of [^18^F]Galacto-RGD (150–200 MBq), a transmission scan was acquired for 5 minutes per bed position (5 bed positions) using three rotating [^68^Ge] rod sources (each with approximately 90 MBq [^68^Ge]). In each subject, a static emission scan was acquired in the caudocranial direction, beginning on average 63.0 ± 4.1 min after injection of [^18^F]Galacto-RGD, covering a field of view at least from the pelvis to the thorax (5–7 bed positions, 5 minutes per bed position).

### Image analysis

Positron emission data were reconstructed using the ordered-subsets expectation maximization (OSEM) algorithm using 8 iteration and 4 subsets. The images were corrected for attenuation using the collected transmission data. The static emissions scans were calibrated to standardized uptake values (SUV). The SUV was calculated according to the following formula: (measured activity concentration [Bq/ml] × body weight [g])/injected activity [Bq]) [41].

In the static emission scans, circular regions of interest (ROIs) with a diameter of 1.5 cm were placed over the left ventricle (for measurement of blood pool activity), the forearm (for measurement of muscle tissue activity) and tumor tissue in three adjacent slices by an experienced operator. Results were expressed in mean SUV. In the tumors, the areas with the maximum intensity were chosen for measurements.

Tumor-to-blood ratios (T/B) and tumor-to-muscle ratios (T/M) were calculated by the following formulas: SUV_tumor_/SUV_blood_ and SUV_tumor_/SUV_muscle_.

In each patient up to 11 lesions were chosen for measurements of SUVs. The maximum number of 11 lesions per patient was chosen in order to avoid a bias by patients with an exceptionally high number of lesions. If there were more than 11 lesions present, e.g. in cases with “superscans”, the lesion with the highest tracer uptake from each afflicted part of the skeleton was chosen. For this purpose, the skeleton was divided into 11 areas: left and right upper and lower extremities; left and right thorax; left and right pelvis; cervical, thoracal and lumbar spine. A volume of interest (VOI) was drawn around each lesion, encompassing the whole lesion. The outer border of each lesion VOI was semiautomatically defined by an isocontour representing 60% of the maximum activity within the VOI. The mean SUV in this VOI was used for further analysis.

For analysis of the detection rate of [^18^F]Galacto-RGD for lesion identification, the number of lesions in each scan, which were identifiable as areas of elevated tracer uptake was noted. The findings of the clinical staging procedures served as standard of reference (including bone scintigraphy in all cases and contrast enhanced CT in 8 cases).

### Statistical analysis

All quantitative data are expressed as mean ± one standard deviation. The correlation between quantitative parameters was evaluated by linear regression analysis and by calculation of Pearson's correlation coefficient R. Statistical significance was tested by using analysis of variance (ANOVA). The correlation between semi quantitative parameters and quantitative parameters was evaluated by the Spearman rank correlation. All statistical tests were performed at the 5% level of statistical significance, using the StatView program (SAS Institute Inc., Cary, NC, USA) or MedCalc (MedCalc Version 6.15.000).
